# An Acute Gangrenous Cholecystitis Caused by Candida auris: A Case From a Greek Hospital

**DOI:** 10.7759/cureus.71338

**Published:** 2024-10-12

**Authors:** Sofia Pouriki, Theoni Agapitou, Aikaterini Tsagkaraki, Panagiota Manthou, Spiridon Tsikrikas, Despoina Varvitsioti, Thomai Kollia, Hariklia Kranidioti

**Affiliations:** 1 Intensive Care Medicine, Center for Respiratory Failure, General Hospital of Thoracic Diseases “Sotiria”, Athens, GRC; 2 Infection Control Office, General Hospital of Thoracic Diseases “Sotiria”, University of West Attica, Athens, GRC; 3 Cardiology, Center for Respiratory Failure, General Hospital of Thoracic Diseases “Sotiria”, Athens, GRC; 4 Intensive Care Medicine and First Department Respiratory Medicine, General Hospital of Thoracic Diseases “Sotiria”, National and Kapodistrian University of Athens, Athens, GRC; 5 Cardiac Intensive Care Medicine, General Hospital of Thoracic Diseases “Sotiria”, Athens, GRC; 6 Second Department of Internal Medicine, National and Kapodistrian University of Athens, Hippocratio General Hospital, Athens, GRC

**Keywords:** antifungal treatment, candida auris, candida-related infections, icu infections

## Abstract

Candida-related infections have increased dramatically in recent years, particularly in severely sick or immunocompromised individuals. Furthermore, the discovery of *Candida auris* in 2009 as a fungus resistant to numerous antifungal treatments has increased its significance. This microorganism is linked to high transmission rates among hospitalized patients, resulting in life-threatening infections and complications. This is a complete case study that explains the reasons and suitable therapy for this medical condition. Despite receiving adequate therapy, individuals with acute gangrenous cholecystitis typically have a poor prognosis. As a result, physicians must be aware of this illness and provide the best therapy as soon as possible. Here, we present a case of gangrenous cholecystitis caused by *Candida auris* in a 58-year-old woman.

## Introduction

Candida cholecystitis infections generally pose life-threatening complications and are associated with risk factors like cholelithiasis, antibacterial therapy, immunosuppression, prolonged ICU stays, malignancy, surgery, and parenteral nutrition [1-3]. The risk factors for *Candida auris* are consistent with other Candida species, reflecting similar clinical presentations. This multidrug-resistant species can be transmitted through contact with contaminated surfaces. The most common invasive infections include bloodstream infections (fungemia), myocarditis, urinary tract infections, surgical wound infections, burn infections, skin abscesses, otitis, meningitis, and bone infections [4,5]. Notably, this study represents an important case of gangrenous cholecystitis as the initial presentation of a *Candida auris* infection. Therefore, it is crucial to identify and manage this potentially fatal fungal infection. Furthermore, existing studies highlight the increased risk of systemic Candida infections, including acute cholecystitis, among diabetic patients with poor glycemic control. Conversely, research by Araujo et al. also indicates that Candida cholecystitis is more prevalent in patients with malignancies [6]. Early detection of an infectious disease epidemic is critical for implementing infection control measures on time and reducing morbidity and mortality. In order to quickly detect *C. auris* during an outbreak in the United States of America (USA), a laboratory created and implemented a novel real-time polymerase chain reaction (PCR) assay [7]. Thus, further research is imperative to establish causative factors and develop appropriate treatments for this specific type of infection.

## Case presentation

A 58-year-old female was transferred to the emergency room (ER) by her family. She was comatose and suffering from an unexplained onset and origin of fever. Her family also noted that the day before her admittance, she experienced dyspnea and vomiting. Her family denied any concerns of chest discomfort, nausea, hearing loss, photophobia, dysuria, or recreational drug usage. The patient was immediately intubated due to concerns over the preservation of their airway. Her medical history showed hemorrhagic stroke with residual symptoms (right hemiplegia), arterial hypertension, obesity, diabetes, and depression. The patient is considered immunocompromised due to diabetes mellitus, for which the patient was not treated properly. She also has a high body mass index (BMI) of 33.

Upon examination, elevated glucose levels (371 mg/dL) and a C-reactive protein level of 4.62 mg/dL were noted. Mild leukocytosis at 10.3k/μL, sodium levels at 134 mmol/L, and a creatinine level of 0.7 mg/dL were also observed, with the rest of her laboratory results falling within normal limits. Brain CT revealed a hypodense area consistent with gliosis in the left occipital lobe, attributed to her prior hemorrhagic stroke, with no additional pathology detected. Chest computed tomography scan (CT) displayed a bilateral mosaic pattern, ruling out pulmonary embolism. The patient did not undergo an abdominal CT. A cardiac ultrasound revealed normal ventricular dimensions, ejection fraction, and vascular status. Subsequently, the patient was admitted to the intensive care unit (ICU).

In the ICU, the patient received sedation, neuromuscular blockade, and initiated treatment with piperacillin/tazobactam (4.5 g four times per day) for seven days because of diagnosed with urine infection with ciprofloxacin resistance. After three days, due to persistent hypoxemia, she underwent prone ventilation, which significantly improved her oxygen exchange and saturation.

**Figure 1:**
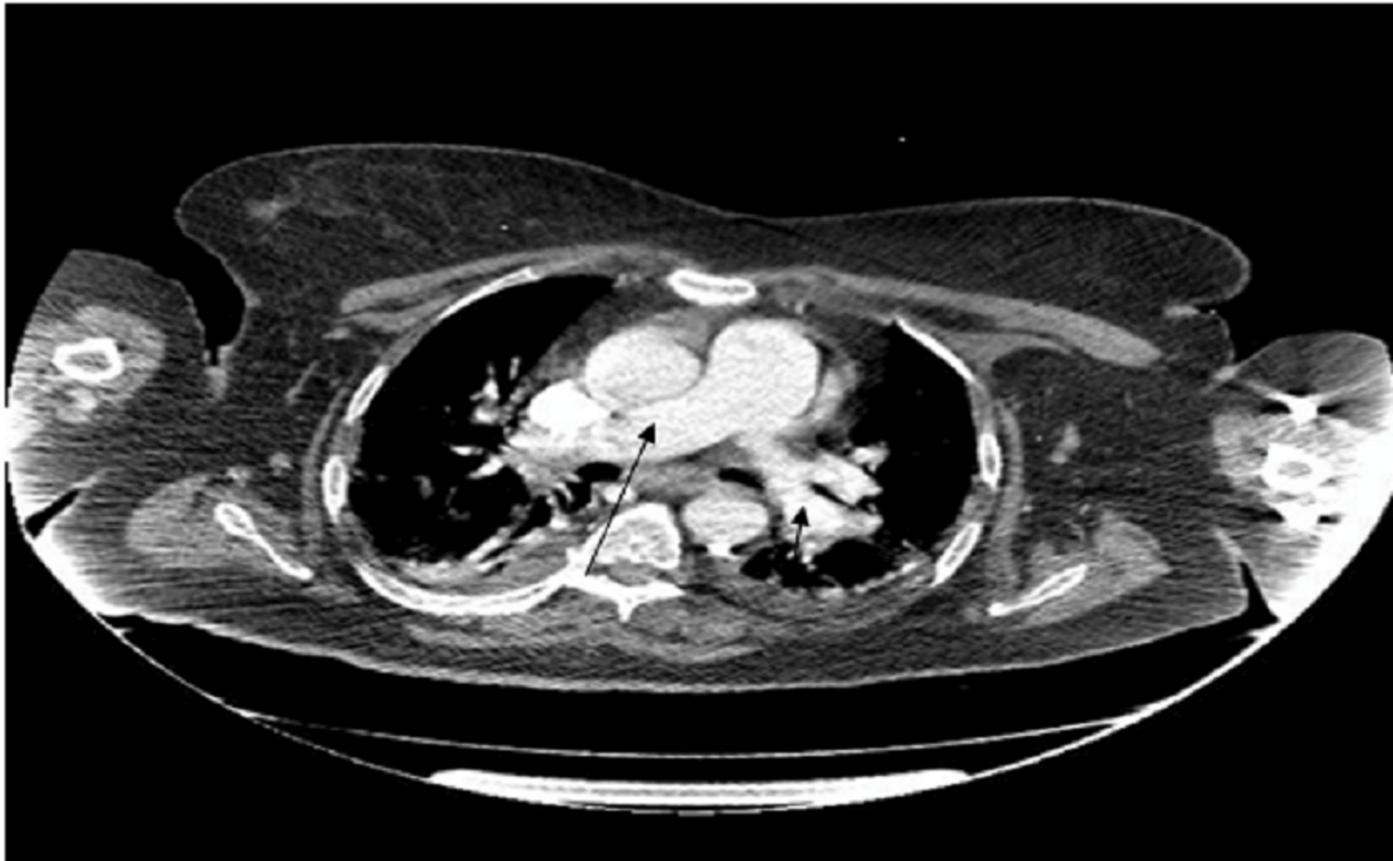
Computed tomography scan of the patient showing a distended gallbladder without pericholecystic edema or biliary dilatation.

Two weeks later, the patient's fever persisted, prompting an infectious assessment. Extensive laboratory tests and body fluid cultures were conducted, all yielding negative results. A lumbar puncture test was performed twice and showed no remarkable findings. On the 20th day of hospitalization, a CT scan revealed a distended gallbladder without pericholecystic edema or biliary dilatation (Figure 1).

**Figure 2:**
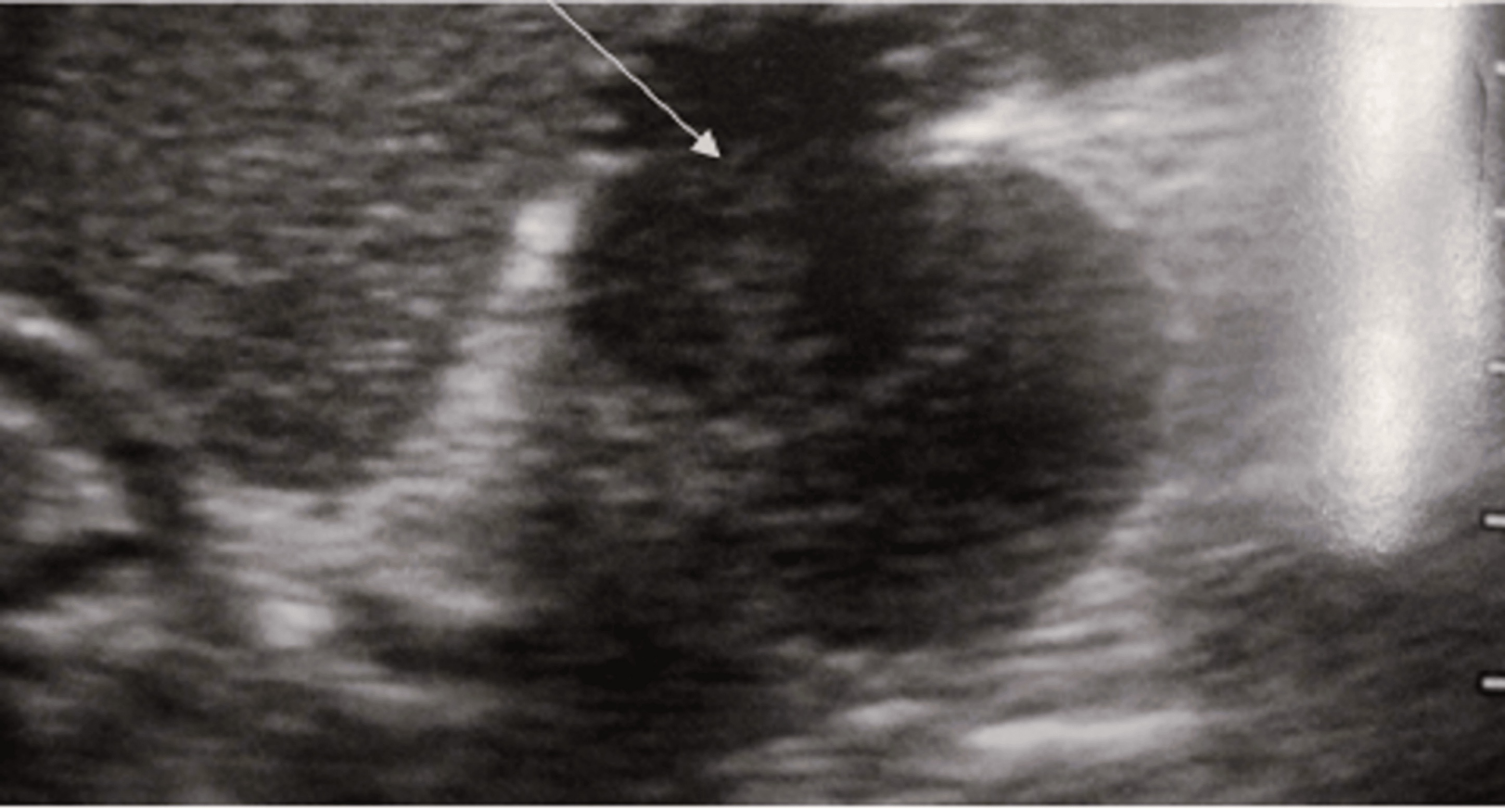
Ultrasound image of the patient showing acute cholecystitis with small gallstones.

While surgical intervention was not recommended, empirical antibiotic and antifungal treatments, alongside parenteral nutrition, were initiated with no observed adverse effects. However, these interventions failed to alleviate her fever. After 12 days, an ultrasound examination identified acute cholecystitis with small gallstones, which was corroborated by an abdominal CT revealing suppurative cholecystitis (Figure 2).

**Figure 3:**
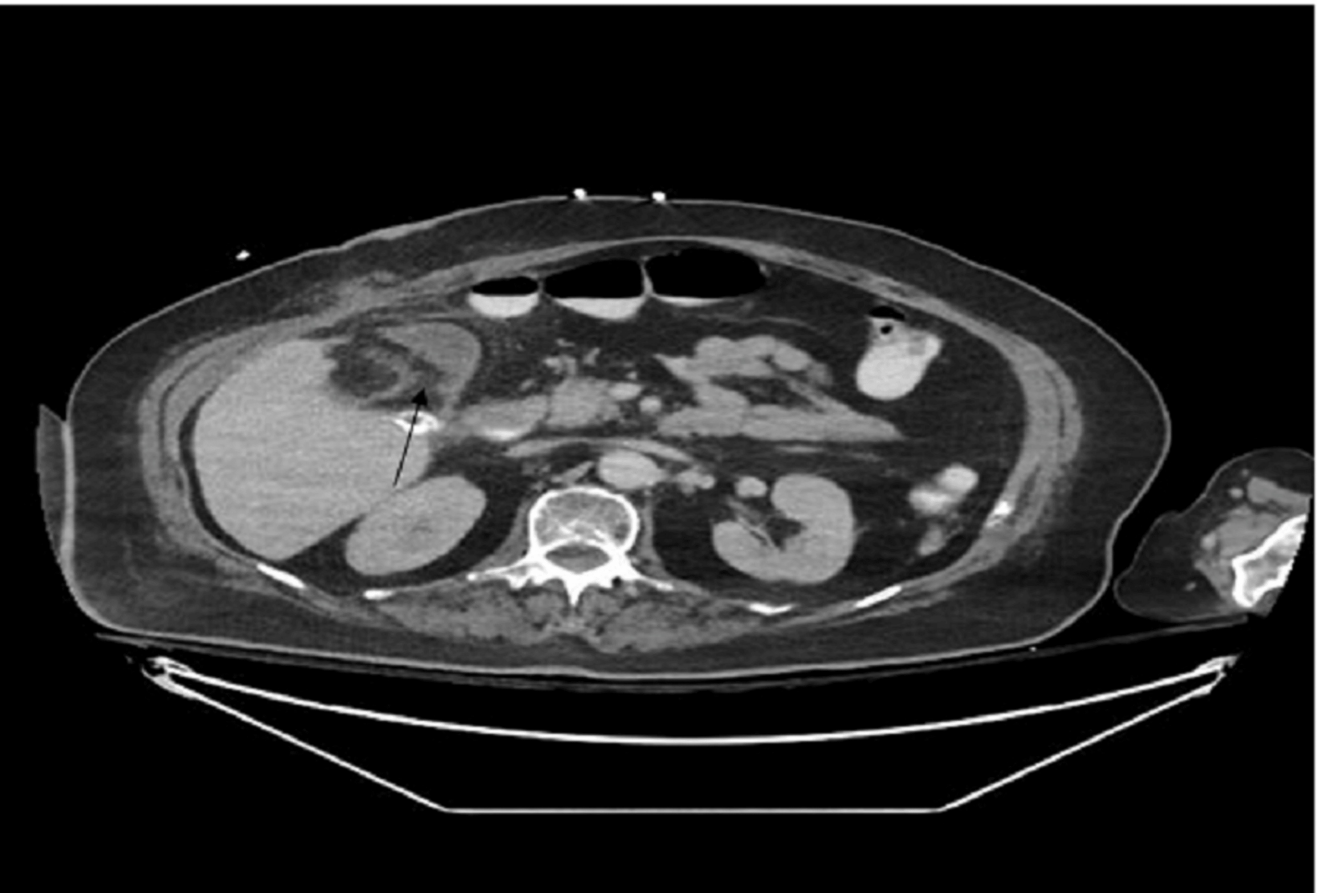
Postoperative CT scan showing findings from the cholecystectomy, including minimal fluid collections in the surrounding area.

Consequently, an open cholecystectomy was performed without complications (Figure 3). After the surgery, the patient had the tracheostomy closed a few days later and was discharged from the ICU without fever. She was breathing spontaneously on a venturi mask delivering 40% oxygen and exhibited good gas exchange. She had a Glasgow Coma Scale score of 15/15, right hemiplegia, and good renal function. The chest imaging did not reveal any pathological findings. Also, the abdominal CT performed postoperatively revealed findings from cholecystectomy and little amount of fluid collection in the area. 

Culture analysis of the purulent material revealed the presence of *Candida auris*. The patient was then commenced on double antifungal treatment, leading to a gradual recovery. Subsequently, she was discharged from the ICU in a stable condition similar to her previous state.

## Discussion

The incidence and prevalence of invasive fungal infections have increased and Candida yeasts are responsible for many severe systemic infections, resulting in increased hospital stays, healthcare costs, and raised mortality [8]. *Candida auris* has been linked to infections of the bloodstream, and urinary and respiratory tracts. In specific conditions like acute cholecystitis, the main cause is the presence of gallstones in the biliary tract. However, in intensive care settings, acute acalculous cholecystitis is not uncommon, accounting for around 2-15% of cases [9], because of several predisposing factors, such as gallbladder epithelial ischemia and reperfusion injury, positive pressure ventilation, parental nutrition, and opioid use [10-12].

Notably, Candida cholecystitis is exceedingly rare [13]. This case represents the first instance of gangrenous cholecystitis caused by *C. auris*. Notably, no cases of gallbladder colonization by *C. auris* have been reported; the majority of *C. auris* invasive infections concern the bloodstream, respiratory and urinary tracts.

For necrotizing cholecystitis, laparoscopic cholecystectomy (LC) is the standard treatment, though complications like bile duct injury can limit its application [14]. Accurate assessment of inflammation severity is vital to select the most appropriate treatment.

The appropriate antifungal agent depends on the virulence of the *C. auris* strain present in the infection. Echinocandin is one of the three antifungal classes with the highest potential against *C. auris*, especially micafungin, making it one of the most indicated drugs for the treatment of infections caused by this pathogen. However, the association with other therapeutic approaches should be considered. If, for example, echinocandin therapy fails or fungemia persists for more than five days, the CDC recommends transitioning to liposomal amphotericin B 5 mg/kg IV once daily. A recent case report involving nine patients with *C. auris* infections in Brooklyn, NY, recommended the use of combination antifungal therapy with echinocandin and liposomal amphotericin B in patients who did not respond to echinocandin monotherapy. This proposal is based on laboratory limitations in terms of rapid *C. auris* detection and susceptibility testing. Amphotericin B is linked to infusion-related events, including nausea, vomiting, chills and rigors, renal failure, and electrolyte abnormalities. All patients, however, should have serum creatinine and electrolyte levels monitored regularly [15,16].

In addition, ibrexafungerp (previously SCY-078), a new antifungal drug under phase III development, has shown strong fungicidal action against *C. auris*. This medication is the first of a new class of structurally unique glucan synthase inhibitors called triterpenoids, and it is accessible in both IV and oral forms. Data presented at IDWeek 2019 demonstrated ibrexafungerp's in vitro and in vivo efficacy against *C. auris*, including multidrug-resistant strains. However, further research including clinical trials is required to reveal the therapeutic potential of new drugs such as SCY-078 as well as the discovery of new drugs in different potential sources, like natural products [17].

## Conclusions

We presented a brief case of a 58-year-old woman who suffered from gangrenous cholecystitis caused by *C. auris*. *Candida auris* can be easily misdiagnosed for other species and treatment is difficult due to multidrug resistance. Clinicians should be aware of this rare pathogen, and it should be treated with appropriate antifungal agents with surgical debridement if needed. More data are also needed to establish the best disinfection and safety protocols to prevent the colonization of this opportunistic pathogen in ICU units and hospital wards.
